# Dietary interventions for managing glucose abnormalities in cystic fibrosis: a systematic review protocol

**DOI:** 10.1186/s13643-018-0757-y

**Published:** 2018-07-18

**Authors:** Laura Birch, Fiona E. Lithander, Simon Langton Hewer, Katie Harriman, Julian Hamilton-Shield, Rachel Perry

**Affiliations:** 1 NIHR Bristol Biomedical Research Centre – Nutrition Theme, Level 3 University Hospitals Bristol Education Centre, Upper Maudlin Street, Bristol, BS2 8AE UK; 2 CF Specialist Centre, Bristol Royal Hospital for Children, Upper Maudlin Street, Bristol, BS2 8BJ UK

**Keywords:** Cystic fibrosis, Cystic fibrosis-related diabetes, Impaired glucose tolerance, Dietary intervention, Glycaemic control, Anthropometry, Body weight, Lung function

## Abstract

**Background:**

Glucose abnormalities in cystic fibrosis (CF) are common, but there is limited evidence to guide their dietary management. Progressive impaired glucose tolerance eventually leads to cystic fibrosis-related diabetes (CFRD), the most prevalent complication of CF, which is associated with increased morbidity and mortality. Optimising glycaemic control improves clinical status and reduces mortality; insulin therapy is the primary means of controlling glycaemia in CFRD, but its role in managing pre-diabetes is less clear. CF dietary therapy requires a high calorie diet due to increased energy expenditure and malabsorption, but this energy-dense diet is typically high in fat and sugar, and high sugar intakes often result in hyperglycaemia in individuals who have impaired glucose handling. Current guidelines for the dietary management of glucose abnormalities in CF are based on clinical consensus rather than empirical evidence. A systematic review conducted in 2012 on the effects of low glycaemic index dietary intervention in CF concluded that there is a dearth of evidence in this area. This review will update the systematic review by Balzer et al. in 2012 and will broaden the scope of their review to include any type of dietary intervention for managing glucose abnormalities in CF.

**Methods:**

Quantitative studies of dietary interventions to manage glucose abnormalities in individuals aged over 5 years with CF and glucose abnormalities will be reviewed. No limits will be placed on language or study design. The comparator will be standard CF dietary therapy (energy dense, high-fat diet) in addition to insulin therapy for individuals with CFRD. Electronic databases will be searched for completed quantitative studies published in peer-review journals that focus on dietary interventions for managing glucose abnormalities in CF. Searches will be conducted from 2000 up to the present day to reflect the evolving improvements in CF management. No restrictions will be placed on study design or language. Duration of the dietary intervention must be a minimum of 2 months and only interventions in out-patient or community settings will be included. Studies must report on dietary intervention, glycaemic control, anthropometry and lung function. Evidence will be assessed for heterogeneity and a narrative review or meta-analysis conducted as appropriate.

**Discussion:**

This systematic review will elucidate current knowledge of the effects of dietary interventions for managing glucose abnormalities in the vulnerable CF clinical population.

**Systematic review registration:**

PROSPERO registration number: CRD42018085569 www.crd.york.ac.uk/prospero/

**Electronic supplementary material:**

The online version of this article (10.1186/s13643-018-0757-y) contains supplementary material, which is available to authorized users.

## Background

There is limited evidence to guide dietary therapy for glucose abnormalities in cystic fibrosis (CF). CF is a chronic, life-limiting, genetic condition, characterised by abnormally thick and dehydrated secretions which lead to organ obstruction, primarily affecting the lungs and the reproductive and digestive systems [1]. Abnormalities in glucose metabolism are common and a spectrum of progressive glucose tolerance abnormalities occur as individuals grow older; indeterminate glycaemia is followed by impaired glucose tolerance (IGT), which affects 20% of 10-year-olds and 82% of the CF population by the age of 30 years [2], before CF-related diabetes (CFRD) eventually develops [3]. CFRD is the most prevalent complication of CF [4], and it is a distinct form of diabetes which occurs in association with pancreatic insufficiency, primarily caused by pancreatic damage. Its pathophysiology is complex and includes the loss of pancreatic islet cells, leading to both insulin and glucagon deficiency, and fluctuating insulin resistance caused by chronic and acute inflammation and infection [4]. CFRD is typically diagnosed in late adolescence or early adulthood [5–7] and affects approximately 20% of adolescents and up to 50% of adults with CF aged over 40 years [4, 5, 8]. The early stages of CFRD is characterised by normal fasting glucose but over time fasting hyperglycaemia develops [8]. The combination of diabetes and CF leads to increased morbidity and is associated with a sixfold increase in mortality [7, 9–11]. Fewer than a quarter of people with CFRD survive beyond the age of 30 years, compared with 60% of CF individuals without diabetes [12, 13].

Optimising glycaemic control is known to improve clinical status and pulmonary function and reduce mortality [5]. Insulin therapy is the primary means of controlling glycaemia in CFRD but its role in -pre-diabetes is less clear [14]. The development of diabetes represents the onset of a second chronic disease, adding further complexity to daily CF treatment regimes. Individuals are required to monitor and control their blood glucose concentrations through regular blood glucose testing and daily insulin injections. Treating IGT in CF with insulin is controversial as it increases patient burden but it is not yet known whether early initiation of insulin reduces morbidity and mortality in the longer term [15].

In cystic fibrosis, a high energy intake is required due to increased energy expenditure and malabsorption, malnutrition risk, and gut abnormalities including delayed gastric emptying, altered intestinal motility and liver disease [7]. To date, there are no meta-analyses or randomised controlled trials of dietary intervention to manage glucose abnormalities in CF, and current guidelines are based on clinical consensus rather than empirical evidence [4, 8]. The goal of CFRD dietary management is to achieve and maintain good nutritional status and normalise blood glucose levels. There are important differences between CFRD and non-CF diabetes, which necessitate a unique approach to diagnosis and management [2]. In practice, due to the lack of specific evidence-based guidelines for managing glucose abnormalities in CF, standard CF nutritional recommendations are frequently applied [4, 8]. However, the energy-dense CF diet is typically high in fat and sugar, and high sugar intakes often results in poor glycaemic control in CF patients with glucose abnormalities. As in any clinical group, patients vary in their specific nutritional needs, and as such, dietary management must be tailored to meet these needs. As practice varies, a consistency of approach and nutritional targeting is needed in this vulnerable group.

Low glycaemic index (GI) dietary intervention has demonstrated benefits in non-CF forms of diabetes, including improved insulin sensitivity, glycaemia and quality of life in both type 1 and type 2 diabetes [16], and it is now recommended as part of their dietary management [17–19]. A systematic review conducted in 2012 to assess understanding of the effect of low GI dietary interventions in young people with CF concluded that there is a dearth of evidence in this area [20]. A scoping search of the literature has revealed that there has been little further research in this field since the 2012 review was conducted. The aim of the current review is to update the 2012 systematic review by Balzer et al. [20], to assess current understanding of glycaemic index dietary intervention in CF and to broaden the scope of their review to include any type of dietary intervention for managing glucose abnormalities in CF. Studies of dietary interventions aimed at improving glycaemic control in individuals over 5 years of age with CF and either IGT or CFRD, will be considered for inclusion. The comparator is standard CF dietary therapy (energy dense, high-fat diet), in addition to insulin therapy for individuals with CFRD. Outcomes will include glycaemic control (primary) and anthropometry and lung function (secondary).

## Methods

This protocol follows the Preferred Reporting Items for Systematic Reviews and Meta-Analyses for Protocols (PRISMA-P) 2015 reporting guideline [21] (see Additional file 1). The review is registered on PROSPERO International prospective register of systematic reviews (www.crd.york.ac.uk/prospero/); registration number: CRD42018085569. If any amendments to this protocol are required when conducting the review, these will be clearly described in the review article when prepared for publication.

### Eligibility criteria

All completed, quantitative studies published in peer-review journals that focus on dietary interventions to manage glucose abnormalities in humans with CF will be included. Due to the evolving improvements in CF management, all articles published from 2000 will be screened to reflect recent care. No limits will be placed on language.

### Participants

Individuals aged over 5 years with CF and either IGT or CFRD  will be included. No restrictions will be placed on upper age, gender, CF mutation or other demographics. Differences in a variety of study level characteristics may result in heterogeneity in the results (e.g. paediatric vs adult patients, good vs poor CF control, transplantation vs no transplantation). Study level characteristics (covariates) that might explain any heterogeneity will be documented and reported.

Glucose abnormalities are defined as follows:

- Oral glucose tolerance test (OGTT):
  *IGT*:
  120-min plasma glucose 7.8–11.1 mmol/l
  OR
  Glycated haemoglobin (HbA1c) ≥ 39 mmol/mol or ≥ 5.7%^1^
*CFRD:*
  fasting plasma glucose concentration ≥ 7.0 mmol/l
  OR
  120-min plasma glucose ≥ 11.1 mmol/l
  OR
  HbA1c ≥ 48 mmol/mol or ≥ 6.5% [22]
  Continuous glucose monitoring (CGM) time above 7.8 mmol/l ≥ 4.5% [23]^2^

All studies in all CF patients will be included, but any additional medication that may affect glycaemia will be acknowledged and if of substantial effect, analysed separately.

### Interventions

Any dietary intervention for managing glucose abnormalities in CF, with or without the use of insulin therapy. Examples include low GI diets and carbohydrate counting. Only dietary interventions of a 2-month duration or more, specifically being used to manage glycaemia in CF, will be included.

### Comparator

There is currently no evidence-based consensus for the dietary management of glucose abnormalities in CF, and thus, the comparators will be standard CF dietary therapy (energy dense, high-fat, high-salt diet [4]) for individuals with IGT and standard CF dietary therapy plus insulin therapy for individuals with CFRD.

### Setting

Diabetes management is primarily an out-patient based speciality and therefore this review will be limited to out-patients and community settings. Any studies solely involving in-patient care will be excluded.

### Outcomes

Primary outcomes are:

- Glycaemic control (OGTT, HbA1c; exploratory outcome: % of CGM time above 7.8 mmol/L)

Secondary outcomes are:

- Body weight (kg)
- Height (m)
- BMI (kg/m^2^)/ BMI-SDS (children & adolescents,)
- Lung function (FEV_1_, FVC)
- Mortality/adverse events
- Adherence and acceptability of dietary intervention

### Study design

A preliminary scoping search of the literature has identified limited evidence in this area. Therefore, all study design types will be included in this review (e.g. RCTs, prospective and retrospective cohort, cross-sectional, case control, case series). Results will be presented according to study design.

### Information sources

Relevant studies will be identified through a comprehensive search of the following main electronic databases: AMED, Embase, MEDLINE via OVID, Web of Science and CENTRAL via Cochrane library. To reflect the evolving improvements in CF management, articles from 2000 up to the present day will be screened, and the first 20 pages of Google Scholar. Reference lists of all included full-text articles will be hand-searched for additional original publications. OpenGrey will also be searched for any additional grey literature. Conference abstracts will be used to help identify potential studies. Conference abstracts from the following major CF and diabetes conferences will be included: International Conference of Cystic Fibrosis, North American Cystic Fibrosis Conference, European Cystic Fibrosis Society, Cystic Fibrosis Foundation Research Conference, American Diabetes Association, European Association for the Study of Diabetes, European Society of Paediatric Endocrinology, International Diabetes Federation. Study authors will be contacted to establish if full-text articles are available or to request any missing data as required.

### Search strategy

**Fig. 1:**
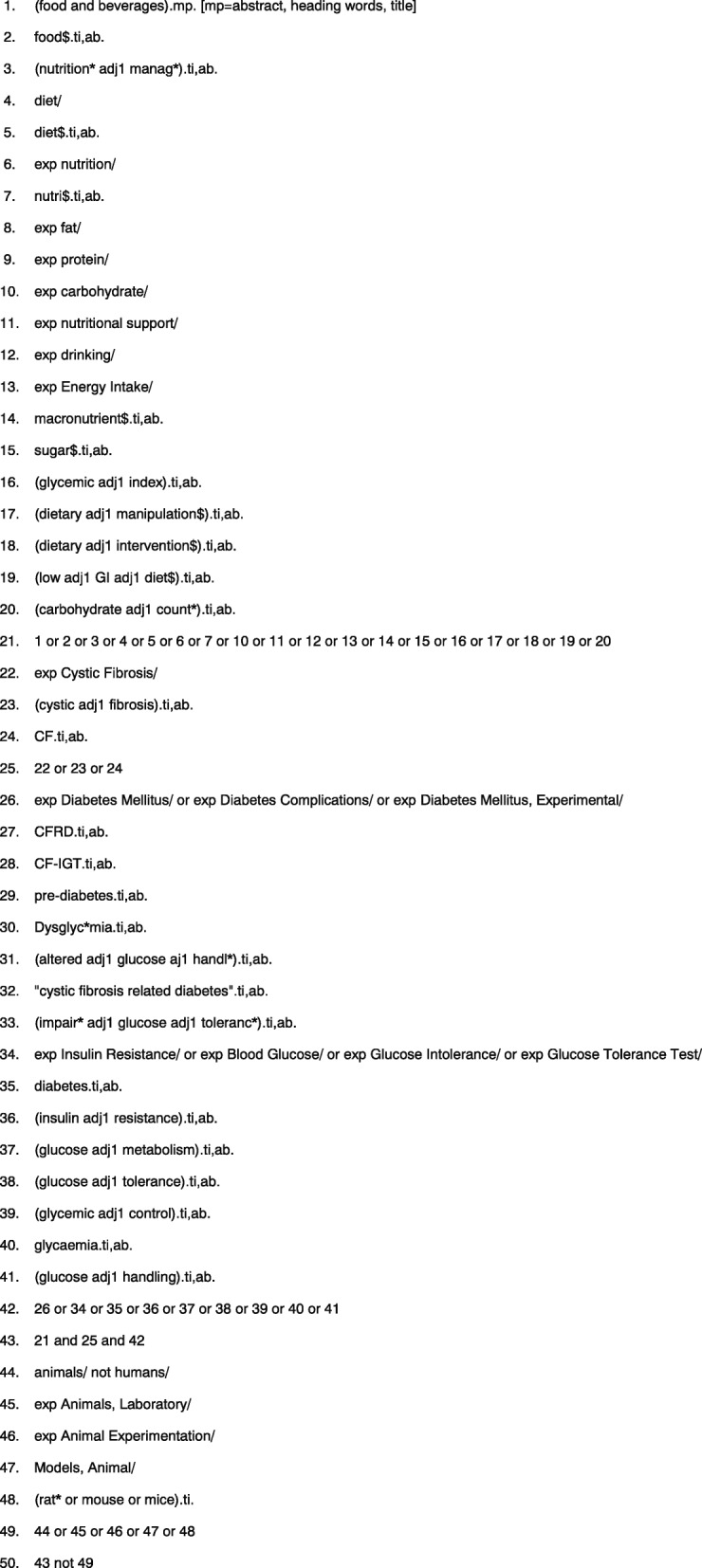
Draft search strategy including planned limits (MEDLINE database)

A draft literature search strategy for MEDLINE database has been developed by the lead author (LB) and an experienced systematic reviewer (RP), using a combination of Medical Subject Headings (MeSH) and keyword terms. This search strategy is presented in Fig. 1. The search strategy will be revised appropriately for each database to take into account differences in controlled vocabulary and syntax rules. A preliminary scoping search of Embase, MEDLINE via OVID and Web of Science databases has been conducted. The search strategy presented in Fig. 1 is a refined version of this preliminary search strategy.

### Data management

EndNote X8.2 reference management software package will be used to manage all the search results throughout the review period. Covidence screening and extraction tool may also be utilised to help manage the review process.

## Selection process

Titles and abstracts will be assessed for eligibility by two members of the review team (LB, RP). Articles that appear to meet the inclusion criteria will be retrieved in full and independently considered for inclusion by two members of the review team (LB, JHS). The reviewers will resolve disagreements in opinion of studies for inclusion through discussion, and the reasons for excluding studies will be recorded.

## Data collection process

Data from full-text articles for inclusion will be extracted independently by two reviewers (LB, RP) using a standardised data extraction template. The template will be piloted by both reviewers before starting the review and modified as required to ensure consistency. The following data will be extracted from each study: study design; sample characteristics (size, inclusion/exclusion criteria, sex, age, socioeconomic status); dietary intervention; results (including but not limited to glycaemic control, anthropometric measures, lung function, adherence and acceptability of dietary intervention); analysis methods; study limitations; and funding sources. Disagreements in opinion of data extracted will be resolved through discussion.

### Risk of bias/quality assessment

Full-text articles of all included research studies will be assessed for methodological quality independently by two members of the review team (LB, RP). The Cochrane Risk of Bias tool [24] will be used for RCTS and ROBINS-I tool [25] for observational studies. Any discrepancies between the two reviewers will be resolved through discussion or third-party adjudication. The quality of all included studies will be assessed using the Grading of Recommendations Assessment, Development and Evaluation (GRADE) system [26].

## Data synthesis

A narrative review of the findings from the included studies will be presented, structured around the outcomes reported. Meta-analysis will be performed only if the identified studies are comparable. The primary outcomes of this review will be continuous variables, and we anticipate that mean values will be reported for all continuous outcomes. Results reporting categorical outcomes will be reported separately. Sub-group analysis based on age (children, adults), glucose abnormality (CFRD, IGT), CF control (good control vs poor control) and transplantation (transplant vs no transplant) will be conducted if there are sufficient numbers of comparable studies. Results will be pooled for comparable sub-sets of studies only where possible.

The preliminary scoping search conducted revealed a relatively small number of studies which appeared to be very diverse. Considerable heterogeneity between studies is therefore anticipated. Where there are sufficient studies deemed comparable, in respect of both the dietary intervention and the outcomes under consideration, their individual effect sizes will be illustrated using forest plots together with combined estimates, using random effect models. Study level characteristics (covariates) that might explain any heterogeneity, such as age, nutritional status and CF control, will be documented and reported. Sub-group analyses as outlined above will be undertaken together with meta-regression if indicated.

## Discussion

This systematic review will elucidate current knowledge of the effects of dietary interventions for managing glucose abnormalities in cystic fibrosis and will highlight areas where further research is needed to inform dietary recommendations for this vulnerable clinical population.

## Additional file

Additional file 1: PRISMA-P 2015 Checklist. PRISMA-P checklist adapted for use with systematic review protocol submissions to BioMed Central journals. (DOCX 33 kb)

## Abbreviations

CF: Cystic fibrosis
CFRD: Cystic fibrosis-related diabetes
GI: Glycaemic index
IGT: Impaired glucose tolerance

## Footnotes

1 The applicability of HbA1c in the CF population is controversial due to increased red blood cell turnover [3] but will be included in this review to be as inclusive as possible.

2 Exploratory outcome is not a validated definition of diabetes
